# Harmonic Scalpel Versus Clips for Ligation of Cystic Duct in Laparoscopic Cholecystectomy: A Systematic Review

**DOI:** 10.7759/cureus.32335

**Published:** 2022-12-08

**Authors:** Chirag Pereira, Shankar Gururaj, Benji Varghese

**Affiliations:** 1 General Surgery, Royal Lancaster Infirmary, Lancaster, GBR; 2 General Surgery, Father Muller Medical College and Hospital, Mangalore, IND; 3 Urology, Wythenshawe Hospital, Manchester, GBR

**Keywords:** bile leak, systematic review, clips, harmonic scalpel, conventional laparoscopic cholecystectomy

## Abstract

The gold standard treatment for benign gallbladder disease is laparoscopic cholecystectomy (LC). LC is commonly performed using monopolar diathermy with ligation of the cystic duct and artery with clips. The aim of the current systematic review was to compare harmonic scalpel (HS) with clips in LC. Eligible studies were identified from PubMed, Cochrane library, Embase and Medline and meta-analysis was conducted using Review Manager 5.4. The primary outcome evaluated was bile leak while secondary outcomes evaluated were overall postoperative complications, operative time, conversion to open surgery and gall bladder perforation. Eight randomized control trials met the eligibility criteria which included a total of 1,205 patients. There was no statistically significant difference between the two groups in terms of bile leak (p = 0.56, I^2^=0%). With respect to the operative time (p = 0.004, I^2^=97%), conversion to open surgery (p = 0.02, I^2^=0%) and gall bladder perforation (p = 0.0001, I^2^=26%) HS was superior to clips. HS is an acceptable alternative to the use of clips when ligating the cystic duct.

## Introduction and background

Laparoscopic cholecystectomy (LC) is considered to be the gold standard treatment for benign gall bladder pathologies [1]. Standard LC involves the use of metal clips to ligate the cystic duct and the cystic artery prior to division with laparoscopic scissors. In some cases, monopolar electrocautery is also used to divide the cystic artery. Monopolar electrocautery still remains the preferred energy device of choice by surgeons while performing LC [2,3]. Alternative methods of bile duct ligation include linear staplers, sutures and endoloops, but these have very seldom been used [4]. The main limitation of the use of monopolar diathermy is that there is a high risk of surrounding thermal damage. Slippage of clips off the cystic duct in relation to the gall bladder causes bile to spill into the surgical field and this invariably causes frequent instrument change which increases the risk of visceral-related injuries [5,6]. A harmonic scalpel (HS) is an energy device that makes use of ultrasound within the harmonic frequency range to coagulate and cut tissue. It is designed to be superior to monopolar diathermy and has the capability to seal vessels 5 to 7mm in diameter. Studies have also shown that thermal energy spread is less with HS as compared to monopolar diathermy [7]. The purpose of the current systematic review is to evaluate the role of HS in successfully sealing the cystic duct in LC as compared to standard clip ligation. The primary outcome evaluated was bile leak while secondary outcomes evaluated were overall postoperative complications, operative time, conversion to open surgery and inadvertent gall bladder perforation during surgery.

## Review

Systematic reviews and meta-analyses were designed and reported according to Preferred Reporting Items for Systemic Reviews and Meta-Analyses (PRISMA) [8].

Search strategy

**Figure 1:**
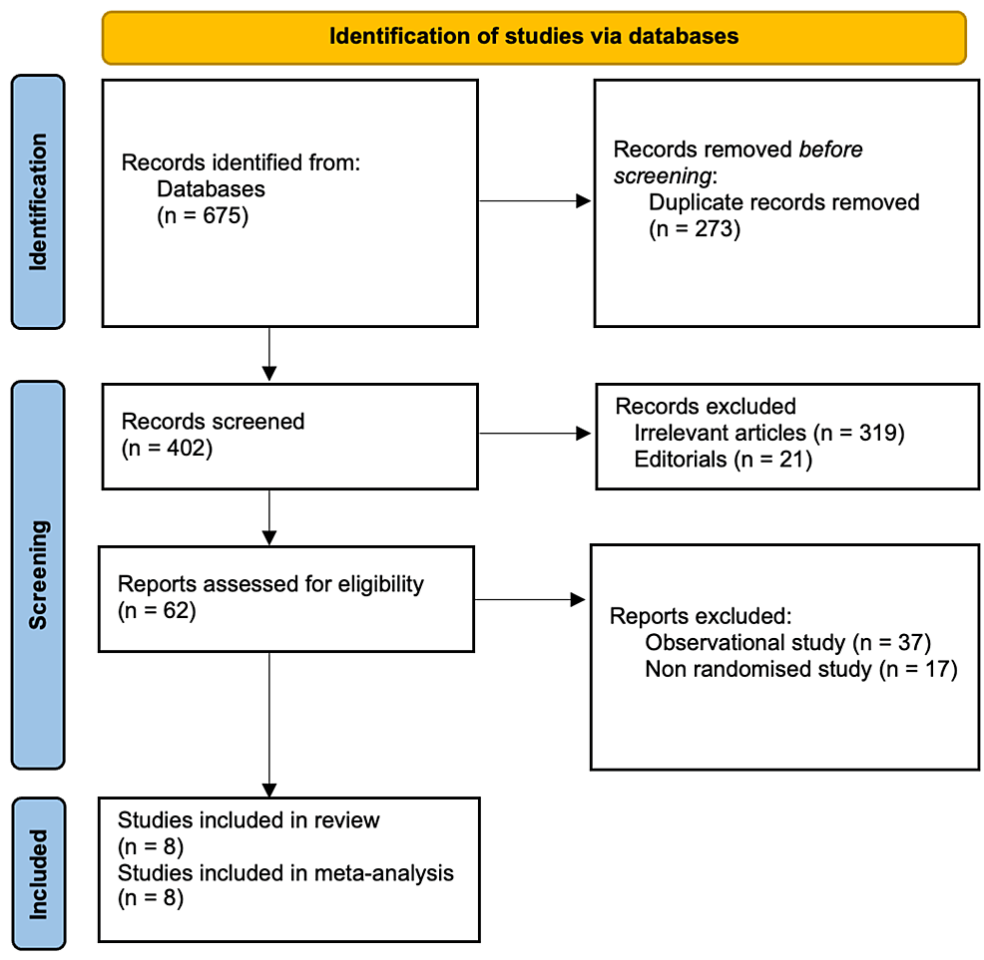
PRISMA flow chart Preferred Reporting Items for Systemic Reviews and Meta-Analyses

A comprehensive literature search was conducted independently by two authors from PubMed, Cochrane library, Embase and Medline. The last date of the search was September 21, 2022. There were no publication date restrictions and only human studies were included. Keywords used for electronic searches were “harmonic scalpel,” “ultrasonic device,” “clips,” “titanium clips,” “cholecystectomy,” “laparoscopy,” “laparoscopic cholecystectomy,” “cystic duct,” “bile leak” and “clipless cholecystectomy.” A flow chart of included and excluded studies is shown in Figure 1.

Study selection

Studies eligible for inclusion were: (1) randomized controlled trials (RCTs) comparing HS with clips in LC, (2) provided information on outcome measures and (3) information on the closure of cystic ducts. Studies excluded were (1) non-RCTs, (2) studies not based on the closure of cystic duct, (3) open cholecystectomies, (4) case reports, observational studies, reviews and meta-analyses.

Data collection

The following data were extracted by two authors from each study: first author, year of publication, country of origin, number of included patients, mean age, gender distribution, operative time, intraoperative gall bladder perforation, type of cystic duct closure used, duration of surgery, postoperative complications, gall bladder perforation and conversion to open surgery.

Assessment of risk of bias

**Figure 2:**
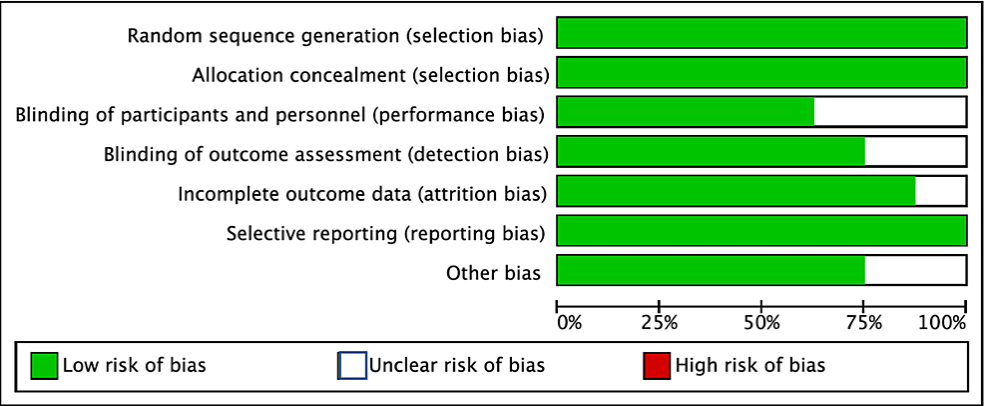
Risk of Bias among included studies

The risk of bias was assessed based on the Cochrane risk of bias tool [9] by two authors. The following categories were classified as low, high or unclear: random sequence generation, allocation concealment, blinding of outcome assessment, blinding of participants and personnel, selective reporting and other sources of bias. The risk of bias was decided by discussion between all authors (Figure 2).

Statistical analysis

Review manager 5.4 was used for statistical analysis. Mean difference was calculated for continuous data at 95% confidence intervals while risk ratio at 95% confidence interval was calculated for dichotomous variables. The Cochrane Q test and I^2^ test were used to assess heterogeneity in the included studies. 0% was considered as no heterogeneity while >50% was considered as significant heterogeneity. Random effect models and fixed effect models were used for analysis appropriately based on the calculated heterogeneity.

Results

Eight RCTs were included in our analysis comprising a total of 1205 patients. There were 547 patients that underwent LC with monopolar diathermy and clip ligation of the cystic duct while 658 patients underwent LC with HS. Characteristics of the clips group and harmonic group are shown in Table 1.

**Table 1:** Characteristics of included studies

Study	Country (Year of publication)	Method of randomization	Clips Group			Harmonic Group		
			Number of patients	Sex Male / Female	Mean Age ± SD (Years)	Number of patients	Sex Male / Female	Mean Age ± SD (Years)
Bessa et al. [10]	Egypt (2008)	Sealed envelope	60	12 / 48	42.5 ± 11.4	60	13 / 47	41.5 ± 10.3
El et al. [11]	Egypt (2010)	Sealed envelope	60	35 / 25	39.93 ± 13.82	60	42 / 18	41.42 ± 10.36
Redwan [12]	Egypt (2010)	Not mentioned	80	33 /47	Not recorded	80	27/ 53	Not recorded
Kandil et al. [13]	Egypt (2010)	Sealed envelope	70	30 / 40	41.38 ± 11.91	70	29 / 41	40.97 ± 11.56
Jain et al [14]	India (2011)	Computer generated random numbers	100	11 / 85	38.67 ± 11.87	100	6 / 90	39.55 ± 11.12
Catena et al [15]	Italy (2014)	Computer generated random numbers	21	10 /11	71.6 ± 6.2	21	11 /10	71.2 ± 7.1
Liao et al [16]	China (2016)	Computer generated random numbers	81	40/41	42.2 ± 10.4	21	51/66	43.4 ± 11.1
Sanawan et al [17]	Pakistan (2017)	Sealed envelope	75	7 / 68	Not recorded	117	22 / 128	Not recorded

**Figure 3:**
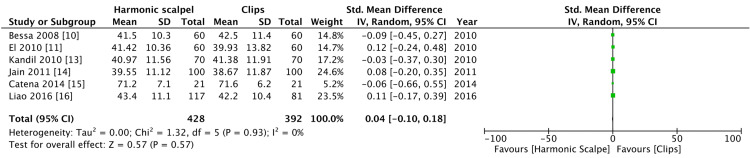
Comparison of age between harmonic group and clips group

There was no significant difference in terms of age and no significant heterogeneity. Two of the included studies [12,17] did not record age as a mean and standard deviation and hence were not included in analyzing forest plots (Figure 3).

Table 2 shows the outcome of the included studies in the clips group and harmonic group.

**Table 2:** Outcomes of included studies

Study	Clips group					Harmonic Group				
	Gall Bladder Perforation	Conversion to open	Operative time (Min)	Bile Leakage	Post op complications	Gall Bladder Perforation	Conversion to open	Operative time (Min)	Bile Leakage	Post op complications
Bessa et al. [10]	20	0	Not recorded	0	4	6	0	Not recorded	0	3
El et al. [11]	11	3	69.71 ± 13.01	2	9	6	2	45.17 ± 10.54	1	5
Redwan [12]	11	0	44.01 ± 6.47	0	Not recorded	8	0	16.8 ± 6.8	1	Not recorded
Kandil et al. [13]	13	2	51.7 ± 13.79	2	11	5	0	33.21 ± 9.62	0	3
Jain et al [14]	18	4	64.70 ± 13.74	0	Not recorded	9	4	50.0 ± 9.35	0	Not recorded
Catena et al [15]	Not recorded	7	106.4 ± 11.3	0	4	Not recorded	1	101.3 ± 10.1	1	5
Liao et al [16]	0	0	51.7 ± 9.6	0	1	0	1	54.9 ± 13.1	1	2
Sanawan et al [17]	16	0	Not recorded	0	Not recorded	5	0	Not recorded	0	Not recorded

Bile leak

**Figure 4:**
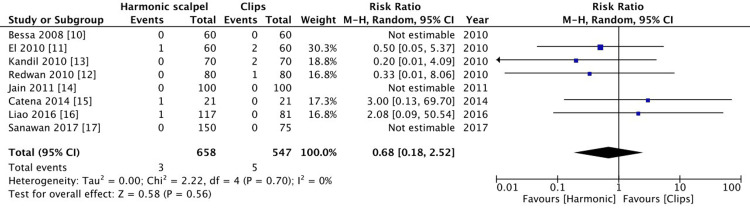
Comparison of bile leak between harmonic group and clips group

The primary outcome of our study was to evaluate bile leak between the harmonic group and the clips group in the early postoperative period (one week following surgery). There were three reported cases of bile leak in the harmonic group and five cases in the clips group. This failed to show a statistically significant difference (p = 0.56, I^2^=0%) (Figure 4).

Operative time

**Figure 5:**
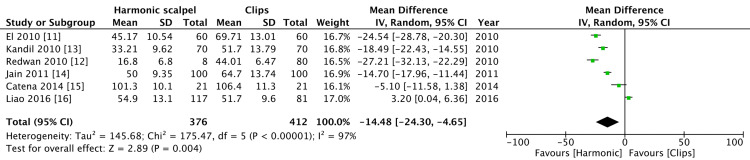
Comparison of operative time between harmonic group and clips group

Operative time was recorded by six studies. Operative time with the harmonic group was shorter as compared to the clips group and this was statistically significant. There was a high level of heterogeneity in regard to operative time (p = 0.004, I^2^=97%) (Figure 5).

Conversion to open surgery and gall bladder perforation

**Figure 6:**
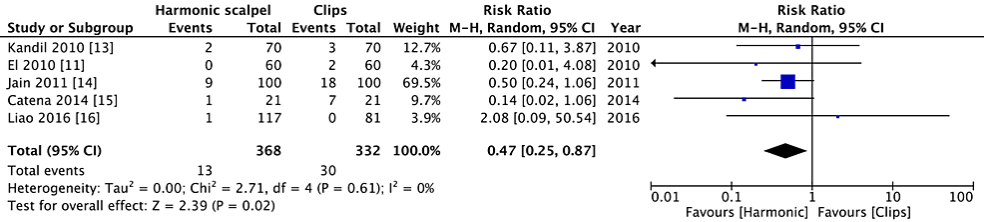
Comparison of conversion to open surgery between harmonic group and clips group

In three of the included studies [10,12,17], there was no conversion of open surgery in either of the groups. There were 13 cases in the harmonic group and 81 cases in the clips group that was converted to open surgery. This was statistically significant with low heterogeneity (p = 0.02, I^2^=0%) (Figure 6).

**Figure 7:**
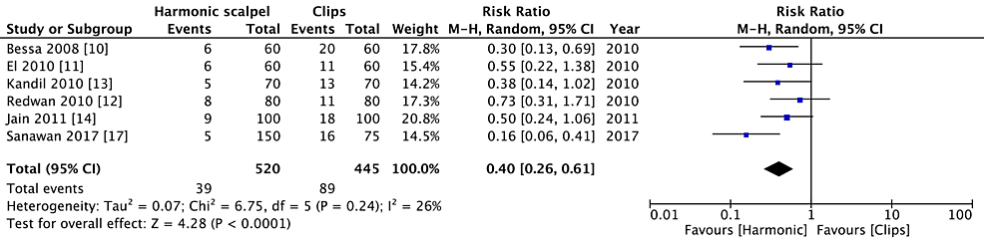
Comparison of gall bladder perforation between harmonic group and clips group

Liao et al. [16] did not have any gall bladder perforation during surgery and Catena et al. [15] had not recorded gall bladder perforation in their study. There were 39 cases of gall bladder perforation in the harmonic group and 89 in the clips group which was statistically significant. There was a low level of heterogeneity (p = 0.0001, I^2^=26%) (Figure 7).

Postoperative complications

**Figure 8:**
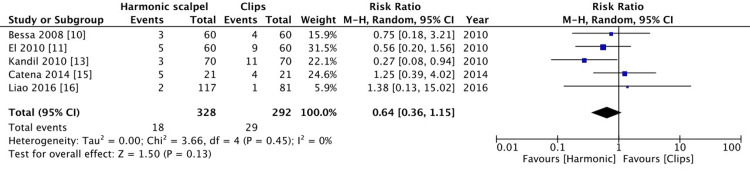
Comparison of operative complications between harmonic group and clips group.

Five studies reported post-operative complications. Since the primary outcome was to evaluate bile leak this has not been included in the analysis as part of post-operative complications. There was no significant difference between the two groups (p=0.13, I^2^=0%) (Figure 8).

Discussion

HS works on the principle of generating high-frequency ultrasound which when applied to tissue produces three main effects of cavitation, coagulation and lastly cutting [18]. Due to these effects, it limits the number of instruments required to perform a safe cholecystectomy. A standard cholecystectomy includes the use of monopolar cautery for dissection, clips for ligation of cystic artery and duct and laparoscopic scissors for division [19]. Since HS can overcome the use of multiple instrumentations, it invariably reduces the chance of intraoperative tissue injury [20]. The heat generated from HS ranges between 60 to 90 degrees Celsius, which is lower as compared to monopolar diathermy which is 150 degrees Celsius [21]. Hence the degree of thermal damage by HS is less as compared to monopolar diathermy.

The main outcome of our systematic review was to evaluate bile leaks following the application of clips versus the use of HS. Although there were a greater number of bile leaks with clip application it was not significant. The most common cause of bile leak is from cystic duct stumps and secondly from inadvertent injury to the biliary tree [22]. Another reason for bile leak is the accessory duct of Lushka, which can leak bile during gall bladder separation from the liver bed [23]. In these situations, HS proves to be quite beneficial.

The main advantage of harmonic seen in this review was that operative time was significantly shorter as compared to the use of clips. When clips are being used in cholecystectomy, it often involves the use of multiple instruments to be taken in and out which adds to the operative time. HS on the other hand acts as a sole instrument to cut and seals tissue at the same time achieving hemostasis [24].

In our review, there were a greater number of cases in the clips group that converted to open surgery as compared to the harmonic group and this was found to be statistically significant and consistent with already published literature [16]. In reference to gall bladder perforation, there were more cases in the clips group where monopolar diathermy was used, and this was consistent with published literature [12-14]. This is most likely to be due to the precision by which HS can be used laparoscopically as compared to monopolar diathermy. Another factor can also be the greater thermal damage caused by monopolar diathermy as compared to HS. Factors such as gall bladder inflammation, operative technique, surgeons' experience, and patient factors such as cirrhosis and obesity will also affect the outcome of open conversion and gall bladder perforation [25].

Limitations to the current review are a smaller size of the included cohort and this can result in a potential publication bias. However, we have chosen to include only RCTs to minimize bias. Data with respect to postoperative complications and gall bladder perforation were not recorded in all studies and this will affect the overall analysis. Studies have not taken patient-related factors into consideration such as but not limited to gall bladder inflammation, obesity, and liver cirrhosis which can potentially affect outcome measures. 

## Conclusions

The current systematic review demonstrated that HS has certain advantages over the use of clips and monopolar diathermy used in conventional LC. With respect to operative time, conversion to open surgery and gall bladder perforation HS proved to be more useful. There was no difference in terms of postoperative bile leak or general postoperative complications. Hence, we conclude that HS is an acceptable alternative to the use of clips when ligating the cystic duct.

## References

[1] Ansaloni L, Pisano M, Coccolini F (2016). WSES guidelines on acute calculous cholecystitis. World J Emerg Surg.

[2] Gelmini R, Franzoni C, Zona S, Andreotti A, Saviano M (2010). Laparoscopic cholecystectomy with Harmonic scalpel. JSLS.

[3] Connor SJ, Perry W, Nathanson L, Hugh TB, Hugh TJ (2014). Using a standardized method for laparoscopic cholecystectomy to create a concept operation-specific checklist. HPB (Oxford).

[4] Nathanson LK, Easter DW, Cuschieri A (1991). Ligation of the structures of the cystic pedicle during laparoscopic cholecystectomy. Am J Surg.

[5] Cirocchi R, Panata L, Griffiths EA (2021). Injuries during laparoscopic cholecystectomy: a scoping review of the claims and civil action judgements. J Clin Med.

[6] Hanazaki K, Igarashi J, Sodeyama H, Matsuda Y (1999). Bile leakage resulting from clip displacement of the cystic duct stump: a potential pitfall of laparoscopic cholecystectomy. Surg Endosc.

[7] Družijanić N, Pogorelić Z, Perko Z, Mrklić I, Tomić S (2012). Comparison of lateral thermal damage of the human peritoneum using monopolar diathermy, harmonic scalpel and LigaSure. Can J Surg.

[8] (2022). Cochrane handbook for systematic reviews of interventions. https://training.cochrane.org/handbook/current.

[9] Higgins JP, Altman DG, Gøtzsche PC (2011). The Cochrane Collaboration's tool for assessing risk of bias in randomised trials. BMJ.

[10] Bessa SS, Al-Fayoumi TA, Katri KM, Awad AT (2008). Clipless laparoscopic cholecystectomy by ultrasonic dissection. J Laparoendosc Adv Surg Tech A.

[11] El Nakeeb A, Askar W, El Lithy R, Farid M (2010). Clipless laparoscopic cholecystectomy using the Harmonic scalpel for cirrhotic patients: a prospective randomized study. Surg Endosc.

[12] Redwan AA (2010). Single-working-instrument, double-trocar, clipless cholecystectomy using harmonic scalpel: a feasible, safe, and less invasive technique. J Laparoendosc Adv Surg Tech A.

[13] Kandil T, El Nakeeb A, El Hefnawy E (2010). Comparative study between clipless laparoscopic cholecystectomy by harmonic scalpel versus conventional method: a prospective randomized study. J Gastrointest Surg.

[14] Jain SK, Tanwar R, Kaza RC, Agarwal PN (2011). A prospective, randomized study of comparison of clipless cholecystectomy with conventional laparoscopic cholecystectomy. J Laparoendosc Adv Surg Tech A.

[15] Catena F, Di Saverio S, Ansaloni L (2014). The HAC trial (harmonic for acute cholecystitis): a randomized, double-blind, controlled trial comparing the use of harmonic scalpel to monopolar diathermy for laparoscopic cholecystectomy in cases of acute cholecystitis. World J Emerg Surg.

[16] Liao G, Wen S, Xie X, Wu Q (2016). Harmonic scalpel versus monopolar Electrocauterization in cholecystectomy. JSLS.

[17] Sanawan E, Qureshi AU, Qureshi SS, Cheema KM, Cheema MA (2017). Effectiveness of ultrasound shear for clipless laparoscopic cholecystectomy versus conventional unipolar electrocautery in patients with cholelithiasis. J Coll Physicians Surg Pak.

[18] Dutta DK, Dutta I (2016). The harmonic scalpel. J Obstet Gynaecol India.

[19] Bittner R (2004). The standard of laparoscopic cholecystectomy. Langenbecks Arch Surg.

[20] Tebala GD (2006). Three-port laparoscopic cholecystectomy by harmonic dissection without cystic duct and artery clipping. Am J Surg.

[21] Koch C, Friedrich T, Metternich F, Tannapfel A, Reimann HP, Eichfeld U (2003). Determination of temperature elevation in tissue during the application of the harmonic scalpel. Ultrasound Med Biol.

[22] Suo G, Xu A (2013). Clipless minilaparoscopic cholecystectomy: a study of 1,096 cases. J Laparoendosc Adv Surg Tech A.

[23] Spanos CP, Syrakos T (2006). Bile leaks from the duct of Luschka (subvesical duct): a review. Langenbecks Arch Surg.

[24] Ai XM, Ho LC, Yang NY, Han LL, Lu JJ, Yue X (2018). A comparative study of ultrasonic scalpel (US) versus conventional metal clips for closure of the cystic duct in laparoscopic cholecystectomy (LC): a meta-analysis. Medicine (Baltimore).

[25] Oymaci E, Ucar AD, Aydogan S, Sari E, Erkan N, Yildirim M (2014). Evaluation of affecting factors for conversion to open cholecystectomy in acute cholecystitis. Prz Gastroenterol.

